# Physiological changes in regional and global left-ventricular strain during exercise - a cardiac magnetic resonance imaging study using tagging and feature tracking in healthy volunteers

**DOI:** 10.1186/1532-429X-18-S1-P59

**Published:** 2016-01-27

**Authors:** Dominik Buckert, Nils Dyckmanns, Wolfgang Rottbauer, Peter Bernhardt

**Affiliations:** Innere Medizin II, Uniklinik Ulm, Ulm, Germany

## Background

Techniques for the evaluation of regional- and global left-ventricular strain parameters are emerging in cardiac magnetic resonance (CMR) imaging. Some of these techniques require dedicated CMR sequences at the time of the imaging examination, others are based on post-processing of routine imaging. Little is known about normal values and range of these parameters, assessed by CMR. Moreover, most studies examined subjects under rest conditions only. Objective of our study was to evaluate a tagging and a feature-tracking approach in a cohort of healthy volunteers under rest conditions and during exercise.

## Methods

Our study cohort consisted of 40 healthy volunteers aged between 19 and 31 years (mean age: 24 ± 3.07 years). CMR imaging was conducted on a 1.5 Tesla whole-body scanner (Intera, Philips Medical Systems, Best, the Netherlands) using a 32-channel phased-array surface receiver coil. To simulate physical exercise, a pharmacological stress protocol based on dobutamine was applied. Standard cine images were assessed using a balanced steady-state free precession (SSFP) sequence. Tagging images were generated using a spatial modulation of magnetization sequence. Cine images were analyzed by the feature tracking module of cvi42, Circle Cardiovascular Imaging, Calgary, Canada. For the evaluation of tagging images, the software solution HARP, Diagnosoft, Durham, USA, was applied.

## Results

Analyzes of tagging images showed a decrease in mean peak circumferential strain (rest: -18.2%, standard deviation (SD): 1.79%; mid stress: -18.41%, SD: 1.90%; max stress: -16.27%, SD: 2.45%), mean peak circumferential strain rate (rest: -98.86%/ms, SD: 24.82%/ms; mid stress: -52.47%/ms, SD: 16.42%/ms; max stress: -19.48%/ms, SD: 156.31%/ms) and mean time to peak circumferential strain (rest: 262.62 ms, SD: 34.54 ms; mid stress: 138.04 ms, SD: 29.59 ms; max stress: 91.78 ms, SD: 8.86 ms) during exercise. Feature tracking analyzes of the same parameters, however, showed an increase over different stages of exercise (mean peak circumferential strain: rest: -19.56%, SD: 2.17%; mid stress: -24.02%, SD: 2.49%; max stress: -24.86%, SD: 1.67; mean peak circumferential strain rate: rest: -184.33%/ms, SD: 43.06%/ms; mid stress: -405.16%/ms, SD: 109.38%/ms; max stress: -614.46%/ms, SD: 167.15; mean time to peak circumferential strain: rest: 325.92 ms, SD: 38.11 ms; mid stress: 227.17 ms, SD: 44.05 ms; max stress: 161.18 ms, SD: 19.38 ms). Method comparison revealed a good agreement between the two imaging modalities though an increasing systematical error occurring at higher levels of exercise (exemplary for mean peak circumferential strain: rest: P for interchangeability: .0008, systematic error: 1.24; mid stress: P for interchangeability: <.0001, systematic error: 5.64; max stress: P for interchangeability: <.0001, systematic error: 8.71).

## Conclusions

We found good agreement between tagging and feature tracking, though there was a remarkable systematic error, especially under exercise conditions.

